# Neural underpinnings of comorbid posttraumatic stress and excessive alcohol use: Opposing effects on loss-related mediofrontal theta in combat veterans

**DOI:** 10.1101/2023.07.12.547253

**Published:** 2023-07-14

**Authors:** Eric Rawls, Craig A. Marquardt, Spencer T. Fix, Edward Bernat, Scott R. Sponheim

**Affiliations:** 1 Department of Psychiatry and Behavioral Sciences, University of Minnesota; 2 Minneapolis Veterans Affairs Health Care System; 3 Department of Psychology, University of Maryland College Park

**Keywords:** PTSD, alcohol, evoked theta, mediofrontal, loss feedback, negative reinforcement

## Abstract

**Objective:**

More than half of US military veterans with posttraumatic stress disorder (PTSD) are dependent on alcohol which complicates efforts to optimize treatment. To understand brain mechanisms that could explain this disabling comorbidity, we investigated how brain responses to negative experiences were related to the clinical symptomatology of PTSD and prevalence of alcohol use).

**Methods:**

We recorded electroencephalography responses to unpredictable gain/loss feedback in veterans who had been deployed to war zones as part of Operations Enduring or Iraqi Freedom (OEF/OIF). We applied time-frequency principal components analysis to event-related potentials (ERPs) to isolate neural responses to gains and losses, identifying a frontal theta-band component reflecting primarily losses (feedback-related negativity, FRN).

**Results:**

The severity of intrusive reexperiencing of traumatic events was associated with enhanced mediofrontal theta signaling during loss, which suggests an increased salience for negative outcomes. At the same time, increased hazardous alcohol use was associated with reduced mediofrontal theta signaling following loss. Thus, enhanced salience signaling during losses was linked to reexperiencing symptoms, while alcohol use may have functioned as a negative reinforcer by maladaptively reducing such salience signaling.

**Conclusions:**

A shared neural mechanism appears to underlie co-occurring posttraumatic stress and hazardous alcohol use and highlights the potential for interventions targeting mediofrontal theta signaling to improve the functioning of individuals experiencing these conditions. Future clinical interventions that target mediofrontal theta might modulate exaggerated negative salience processing and effectively reduce trauma-related psychopathology and the draw of alcohol consumption.

## 1 Introduction

Veterans returning from combat deployments are at risk for mental health problems including posttraumatic stress disorder (PTSD) and alcohol dependence (AD). Approximately 23% of veterans returning from recent military deployments met DSM-IV-TR criteria for PTSD (1), while approximately 10.5% met criteria for AD (2). The principal symptoms of PTSD include intrusive reexperiencing of traumatic event(s) that interferes with daily functioning, avoidance of everyday reminders of trauma, disturbances in cognition and mood, and hyperarousal symptoms such as heightened vigilance of one’s surroundings and self-reported enhancement of startle responses (3). There is a critical but understudied connection between PTSD and AD. Fifty to seventy five percent of veterans with PTSD meet diagnostic criteria for AD, and having received a PTSD diagnosis increases the odds of receiving an AD diagnosis 4-fold (2). In this study, we investigated the co-occurrence of PTSD and AD using measures of brain activation related to experiencing salient outcomes that were either negatively or positively valenced.

Recent perspectives have emphasized the application of predictive coding theories (4) to understand the etiology of PTSD (5,6). Predictive coding suggests that a primary function of the brain is to generate predictions about incoming sensory information (called “priors”), and to update these priors when incoming sensory information does not match the brain’s predictions. The application of predictive coding to PTSD suggests that traumatic experiences can lead the brain to begin defaulting to overly strong priors about the potential of threats and danger, contributing to a heightened processing of stimuli as salient and indicative of negative consequences (i.e., negative salience processing). Heightened negative salience processing could be especially linked to intrusive reexperiencing of traumatic events (5), as typically benign stimuli trigger the brain to generate strong predictions of threat that are tied to past experiences and difficult to update or suppress. It is possible that this also provides a mechanistic explanation for the prevalence of heavy alcohol use in PTSD, as chronic alcohol use could reduce the intensity of the brain’s negative predictions, providing negative reinforcement by promoting alcohol use through diminishing the degree of perceived threat posed by stimuli. Negative reinforcement can be defined as the process by which avoidance or escape from aversive states subsequently increases a behavior. In this case, we hypothesize that the behavior of drinking could be negatively reinforced because alcohol could lead to reduced intrusive reexperiencing symptoms, which may provide short-term relief but lead to addictive behaviors due to strengthening of drinking behaviors.

To probe neural circuitry that processes salient negative outcomes and may be relevant to symptoms of PTSD and AD, we recorded electroencephalography (EEG) responses to unpredictable gain/loss feedback. The EEG response to gain/loss feedback includes a mediofrontal feedback-related negativity (FRN) emerging 250–300 ms following feedback, which is more negative for loss than for gain outcomes (7). The FRN corresponds with activity in the theta frequency band (4–8 Hz) and likely indexes activation of core regions of the salience network in the brain (8), such as the anterior cingulate cortex (9). As a signal reflecting both negative emotion and cognitive control, theta-FRN could reflect the effects of posttraumatic stress on the brain’s salience systems as well as the negatively reinforcing effects of alcohol use. Additionally, the feedback-locked ERP contains a reward positivity (RewP) with a broad central-parietal topography and timing like the P300. The RewP is greater for gain than loss outcomes (10), contains mostly delta-band activity (0.5–3 Hz), and is associated with externalizing personality traits (11), which include behavioral impulsivity and aggressiveness (12). The P300 is thought to reflect activation of frontoparietal executive networks of the brain (13) and has a strong genetic basis that reflects a predisposition toward maladaptive substance use (14–16). Delta-RewP may reflect a moderately specific neural predisposition for alcohol use rather than the emotional distress associated with PTSD.

In AD, diminished salience of loss and gain is evident in the smaller FRN and RewP (17), while in PTSD, hyperarousal is associated with enhanced RewP (18). Yet, it is unknown how PTSD in conjunction with AD affects brain processes tied to loss and reward. In the present study, we used a gambling task to examine processing of gain/loss outcomes related to symptoms of PTSD and AD. Briefly, intrusive reexperiencing PTSD symptoms and heavy drinking showed opposing associations with loss salience processing as indexed by theta-FRN activation. We interpret our results in light of predictive coding (4–6), suggesting trauma results in overly strong predictions of threat, leading to enhanced salience signaling for aversive outcomes. Findings are consistent with a model where heavy drinking might provide negative reinforcement by reducing heightened salience signaling characteristic of intrusive reexperiencing.

## 2 Methods & Materials

### 2.1 Participants

The sample consisted of 128 US military veterans who had been deployed to Operations Iraqi Freedom or Enduring Freedom (see Table 1 for demographics). Recruitment targeted veterans with likely posttraumatic stress disorder (PTSD) diagnoses as well as non-treatment-seeking veterans with similar deployment experiences [see (19) for complete recruitment information]. Study procedures were approved by the Institutional Review Boards at the Minneapolis Veterans Affairs Health Care System and the University of Minnesota, and study participants completed a written informed consent process prior to undergoing the study procedures. We assessed sex assigned at birth and gender identity using self-report; both were concordant for the entire sample. We assessed racial/ethnic identity using self-report. No prior publications have involved the data collected using the gambling paradigm that is the focus of this manuscript (7).

### 2.2 Clinical Assessment

Trained and supervised interviewers conducted assessments for psychopathology using the Structured Clinical Interview for DSM-IV Axis I Disorders [SCID-I; (20)]. Consensus teams including at least one licensed doctoral-level clinical psychologist reviewed all available research and clinical information to generate consensus diagnoses which included PTSD, subthreshold PTSD, and alcohol dependence (AD). Individuals were given a subthreshold PTSD designation if they endorsed at least one symptom in each DSM-IV-TR symptom grouping for PTSD, consistent with rating schemes meant to increase sensitivity for clinically meaningful presentations of PTSD symptoms (21). Interviewers characterized posttraumatic stress symptoms using the Clinician-Administered PTSD Scale for DSM-IV [CAPS-IV, fourth edition; (22,23)]. We subdivided the CAPS-IV into four subscales based on previous meta-analytic research on the factor structure of the CAPS-IV (3,24,25), which provided measures of the severity of intrusive reexperiencing (B1 - B5), avoidance (C1, C2), dysphoria (C3 - D3), and hyperarousal symptoms (D4, D5). Participants only completed the full CAPS if they met criteria A1/A2 and B of the CAPS using DSM-IV-TR criteria (i.e., endorsed a traumatic event with an intense emotional response and later experienced intrusive reexperiencing); as such, dimensional analyses included a subsample of n=82 subjects who reported a traumatic event with current reexperiencing (26).

We assessed the severity of alcohol use with the Alcohol Use Disorders Identification Test (AUDIT)-C (27), a 3-item self-report measure of frequency of alcohol use, amount of alcohol use, and frequency of binge drinking. The scale has a maximum score of 12, and the cutoff for clinically meaningful drinking is a score of 4 for men or a score of 3 for women. We assessed for a history of mild traumatic brain injury (mTBI) using the semi-structured Minnesota Blast Exposure Screening Tool (MN-BEST; Nelson et al., 2011), focusing on the three most severe self-identified deployment-related blast exposure events. We achieved consensus on mTBI via assessment teams that included at least one licensed clinical neuropsychologist. Importantly, the study recruitment criteria used a diagnosis of pre-deployment psychopathology as part of exclusion criteria, thus the clinical presentations of psychopathology assessed in the present study are likely to have been acquired post-deployment [see (19)].

### 2.3 Gambling Task

Participants completed a gambling paradigm (7). Each trial offered participants a two-option forced choice. Options were 5 or 25 points, and could be paired in any fashion (i.e., 5/5, 5/25, or 25/25). Choices were presented within black squares which remained on the screen until participants selected one option. One hundred ms following the choice, each square turned red or green (Figure 2A). If the chosen option turned green, the indicated amount was added to the participant’s running score. If the chosen option turned red, the indicated amount was instead subtracted from the participant’s running score. The color of the unchosen option also changed, to indicate what the outcome would have been if the participant had instead chosen that option. Participants completed 256 trials, divided into 8 blocks with self-paced breaks in between. This task required approximately 20 minutes to complete. An important feature of the task was the unpredictable nature of choice feedback. The primary behavioral outcome was risky choice proportion, defined as the percentage of times a participant chose the ‘25’ option when presented with a choice between ‘5’ and ‘25.’ This risky choice proportion was calculated separately for trials following gains and losses Participants are often more risk prone following losses compared with gains (7).

### 2.4 EEG Acquisition and Preprocessing

EEG was sampled at 1024 Hz using a 128-channel BioSemi ActiveTwo EEG system, acquired reference-free (via CMS/DRL sensors). EEG data were preprocessed as described in (11). Trial-level ERPs were epoched (−1,000 to 2,000 ms) and baseline corrected for the 150 ms preceding feedback stimulus presentation. Ocular artifacts were removed using a regression-based method (29), as implemented in the EMCP software program. A careful visual inspection of the data was undertaken to identify and exclude additional movement and other artifacts to minimize their impact on the time-frequency PCA decomposition. Exclusionary criteria were also applied. First, to exclude ocular artifacts remaining after ocular correction, trials on which activity at frontal electrode sites C12 or C25 exceeded 100 μV within a 1,500-ms poststimulus window (relative to median activity within a 800-ms window immediately preceding the stimulus), and vice versa (preceding 800-ms activity, relative to the median of the 1,500-ms poststimulus ERP window), were excluded from further processing. Additionally, any electrodes that became disconnected during the procedure were interpolated.

### 2.5 Time-Frequency PCA Analysis of Event-Related Potentials

**Figure 1.**
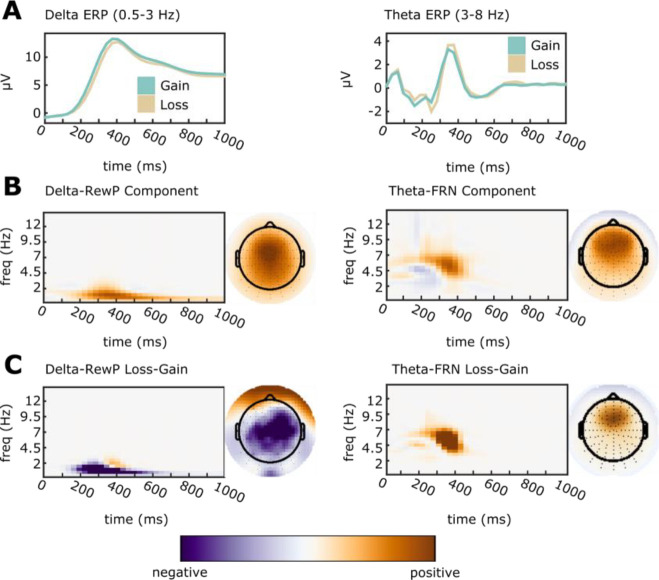
Time-Frequency Principal Components Analysis was applied to separate overlapping ERP activation. All TF surfaces and topoplots are plotted with zero (white) as midpoint. Data units are arbitrary since plots depict PC-weighted power; thus, each plot is scaled to the range of the data. A: Averaged ERP waveforms were filtered into delta (0.5–3 Hz; Cz electrode) and theta (3–8 Hz; FCz electrode) bands. B: ERP waveforms were decomposed, and components reflecting the delta-RewP and theta-FRN response were selected for further analysis based on their PC weights. Components were selected for analysis based on an average over gain/loss conditions. C: To confirm the selected components, we calculated topographic maps and time-frequency surfaces for the average subtraction of loss-gain loadings. As expected, delta-RewP showed greater activation for gains than for losses (left panel), while theta-FRN showed greater activation for losses than for gains (right panel).

We reduced the dimensionality of ERPs during gain/loss processing using time-frequency principal components analysis [tf-PCA; (30,31)]. First, we calculated ERPs at each scalp site, separately for gain/loss trials. To allow the tf-PCA to define the boundary between delta and theta, we pre-filtered each subjects ERP waveforms using a 4-Hz low-pass Butterworth filter for delta, and 2-Hz high-pass Butterworth filter for theta (third order, zero-phase). Each filtered ERP waveform was then transformed into a time-frequency (TF) representation using the reduced interference distribution (RID) with binomial kernel (32). We vectorized TF surfaces (per subject) and generated a data matrix of dimensions subjects-by-TF points. We then reduced dimensionality of this matrix using PCA applied to the covariance matrix. We chose the number of components to retain using a scree plot of the eigenvalues, and by comparing tfPCA loadings and scalp topographies to prior applications of tfPCA in this paradigm, e.g. (11), and consistent with recommendations (31). For delta frequencies, we retained a single component which explained 62% of variance. For theta frequencies, we retained three components which explained 22, 21, and 9% of variance respectively. We rotated the resulting component loadings using a varimax rotation as described by (11,30). Vectorized component loadings were then reassembled into time-frequency matrices and interpreted. The delta-band component loadings mapped well onto the scalp distribution and timing of the centro-parietal P300/RewP. The first theta-band component appeared to reflect the N1 ERP component, with maximal loadings over occipital sensors PO7/PO8, frequencies ~4–8 Hz, and time periods ~150 ms post-feedback. The second theta-band component appeared to reflect the FRN, with expected maximal loadings around mediofrontal sensor FCz, frequencies ~5 Hz, and time periods ~350 ms. The third theta-band component reflected an ongoing ~3 Hz oscillation maximal at mediofrontal sensors. Our primary analyses focused on the single delta-band P300/RewP component and the second theta-band FRN component as identified by the separate tf-PCAs. Dependent variables were calculated as the average across the respective PC-weighted TF surfaces. We examined delta activity at sensor Cz and theta activity at sensor FCz, where component scalp distributions were maximal. TF-PCA analysis and components are summarized in Figure 1.

### 2.6 Statistical Analysis Strategy

We analyzed risky choices and tfPCA delta/theta component activation using robust linear mixed-effects models (rLMMs) fit with the ‘robustlmm’ package, version 3.0–4 (33). We used rLMMs because PC-weighted theta (FRN) and delta (RewP) were highly skewed (skewness = 1.9 and 1.7 respectively) relative to the assumptions of non-robust LMMs (34). We estimated robust LMM *p*-values using robust *t-*statistics and Kenward-Roger approximated degrees-of-freedom obtained from a standard LMM fit with the ‘lme4’ package (35). Robust LMMs predicted risky choice proportion and delta/theta PC-weighted ERP activation using a single within-subject factor of Outcome (gain/loss). Models included two or more between-subjects factors describing clinical presentation. First, for each dependent variable (DV; behavioral risky choices, theta-FRN, delta-RewP) we ran a model with between-subjects factors based on clinical diagnoses of PTSD, mTBI, and AD (entered as simultaneous predictors). Then, for each DV we ran an additional model that used the four CAPS subscales (intrusion/ avoidance/ dysphoria/ hyperarousal, individually z-scored across participants), alcohol use severity (AUDIT-C; z-scored across participants), and blast mTBI severity (MN-BEST; z-scored across participants) entered as simultaneous predictors. We examined the association of risky choices with brain indices of reinforcement processing using a rLMM that predicted risky choice proportions with a categorical factor of Previous Outcome (Previous Gain/ Previous Loss) and including delta-RewP and theta-FRN as continuous predictors. All models contained a random intercept per participant and interaction terms between the within-subjects Outcome factor and all between-subjects factors but did not include interactions of between-subjects factors. Given the wide age range of our sample (22 – 59 years old), we screened our three DVs for significant associations with age. Theta-FRN showed a significant association with age which was not of interest to the current investigation (*r* = −.25, *p* < .001); thus theta-FRN analyses included age as an additional covariate. Significant categorical-categorical interactions were characterized using emmeans, and significant categorical-continuous interactions were characterized using emtrends function, both implemented in the R ‘emmeans’ package, version 1.7.4–1 (36).

## 3 Results

### 3.1 Risky Gambling Behavior is Related to Alcohol Use

**Figure 2.**
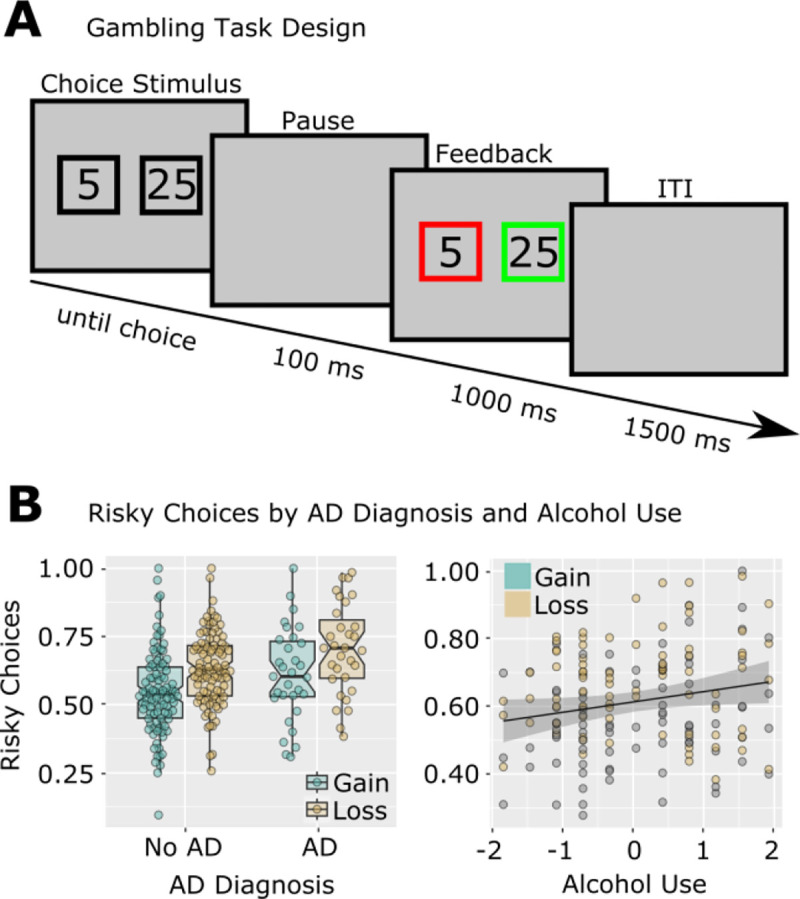
Risky Gambling Behavior is Related to Alcohol Use in Previously Deployed Veterans. A: Design of the modified gambling task. B: Risky choices were increased following losses compared to gains. Individuals with AD and with higher AUDIT-C scores made more risky choices. Note that individual data points are shown to differentiate gain/loss observations, but all statistics were main effects over both Gain/Loss conditions (thus there is only one regression line, rather than separate regressions for gain and loss). AUDIT-C was standardized for analysis and plotting; risky choice proportions were standardized for analysis but not for plotting.

A diagram of the gambling task and of risky choice rates is shown in Figure 2. Risky choice behavior on the gambling task showed an expected main effect of Outcome (gain/loss) in all analyses, *ts* > 7, *ps* < .001, indicating higher risky choice behaviors following loss outcomes. Diagnosis-based analyses showed no effects of PTSD or mTBI, but revealed a main effect of an AD diagnosis, *t*(124) = 2.34, *p* = .02, indicating overall higher risky choice behavior in participants with AD. Likewise, an analysis of symptom severity revealed a main effect of AUDIT-C, *t*(75) = 2.03, *p* = .04, indicating overall higher risky choice behavior in participants with greater alcohol consumption. This analysis failed to show any effects of PTSD symptomatology or mTBI severity on risky choice behaviors.

### 3.2 Delta-RewP is Related to Alcohol Use

**Figure 3.**
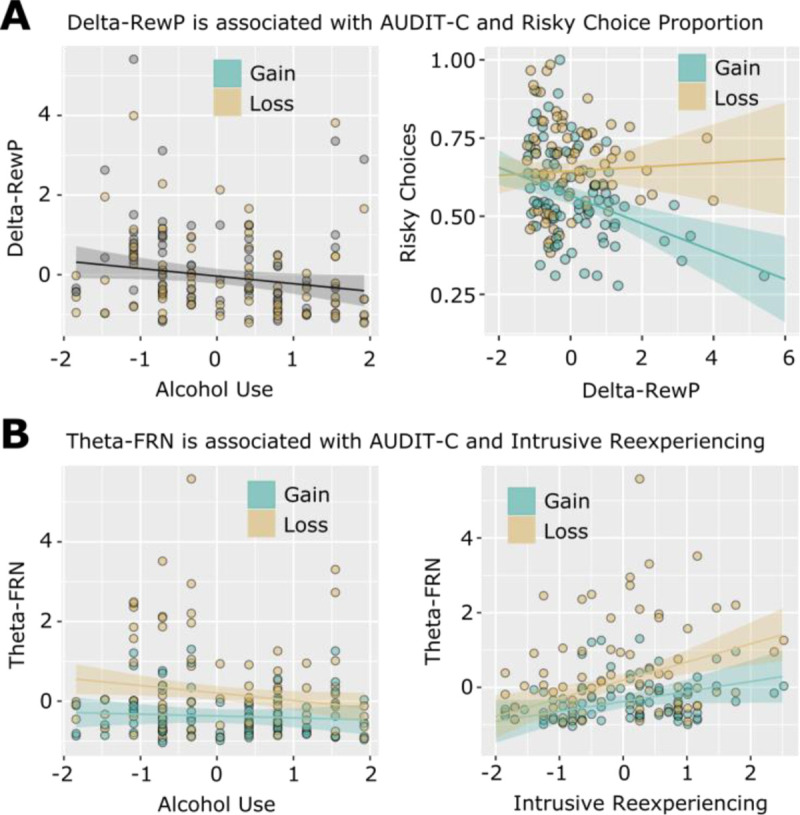
Delta and theta feedback components are related to alcohol use, intrusive reexperiencing, and risky choices in combat veterans. A: Delta-RewP activation was negatively associated with AUDIT-C scores and with risky choices following gains. Delta-RewP and Alcohol Use (AUDIT-C) were standardized for analysis and plotting; risky choice proportions were standardized for analysis but not for plotting. Note that for the left panel, individual data points are shown to differentiate gain/loss observations, but statistics indicate a main effect over both Gain/Loss conditions (thus there is only one regression line, rather than separate regressions for gain and loss). B: Theta-FRN activation was associated with less severe alcohol use (AUDIT–C scores), and more Intrusive Reexperiencing symptoms related to traumatic events. Theta-FRN, Intrusive Reexperiencing, and Alcohol Use (AUDIT-C) were standardized for analysis and plotting.

Our analysis of time-frequency delta PC-weighted activation (i.e., the centro-parietal delta-band activity underlying the RewP) demonstrated a main effect of Outcome for all analyses, *ts* > 3, *ps* < .002, indicating relatively greater activation for gains compared to losses. This component showed no relationship to any categorical diagnosis (PTSD/mTBI/AD). However, analyses of symptom severity revealed a significant main effect of AUDIT-C total score, *t*(75) = −2.01, *p* = .048, indicating decreasing delta-RewP activation with increasing hazardous drinking, standardized AUDIT-C fixed effect estimate = −.19, 95% CI = [−.38, −.001]. There were no effects of continuous measures of PTSD or blast-related mTBI severity. Thus, this analysis revealed that blunted delta-RewP activation was related to increases in hazardous drinking but was unrelated to PTSD or mTBI (Figure 3A).

### 3.3 Opposing Effects of Intrusive Reexperiencing and Alcohol Use on Theta FRN

Our analysis of time-frequency theta PC-weighted activation (i.e., the mediofrontal theta-band activity underlying the FRN) demonstrated a main effect of Outcome for all analyses, *ts* > 8, *ps* < .001, indicating greater activation for losses than gains. Our analysis of individual differences using diagnoses of PTSD, mTBI, and AD failed to reveal effects. However, our analysis of individual differences using dimensions of PTSD symptoms, alcohol use, and blast-related mTBI yielded a main effect of Intrusive Reexperiencing, *t*(75) = 2.93, *p* = .004. The main effect of Intrusive Reexperiencing was qualified by a significant interaction with Outcome, *t*(75) = −2.09, *p* = .04. Finally, the model also simultaneously identified a significant interaction between AUDIT and Outcome, *t*(75) = 2.09, *p* = .04. Post hoc examination revealed that greater Intrusive Reexperiencing severity was associated with enhanced theta activation during loss conditions, standardized fixed-effect estimate = 0.46, 95% CI = [0.20, 0.71], *t*(104) = 3.52, *p* < .001, but not gain conditions, standardized fixed-effect estimate = 0.24, 95% CI = [−0.02, 0.49], *t*(104) = 1.94, *p* = .07 (Figure 3b). Post hoc examination of the significant AUDIT-Outcome interaction indicated that more alcohol use was associated with reduced theta activation during loss conditions, standardized AUDIT fixed-effect estimate = −0.19, 95% CI = [−0.35, −0.03], *t*(104) = −2.29, *p* = .02, but not gain conditions, standardized AUDIT fixed-effect estimate = −0.05, 95% CI = [−0.21, 0.11], *t*(104) = −0.60, *p* = .55 (Figure 3B). This analysis revealed no effects of blast-related mTBI severity. As such, loss processing as embodied in frontal midline theta is simultaneously linked in opposing ways to the severity of PTSD-related intrusive reexperiencing and elevated hazardous alcohol use in previously deployed combat veterans.

### 3.4 Delta-RewP, but not Theta-FRN, is Related to Risky Choice Behavior

As previously noted, risky choice behavior on the gambling task showed an expected main effect of Outcome (gain/loss) in all analyses that indicated higher risky choice behaviors following loss outcomes. We next examined whether these risky choice behaviors following gains and losses were differentially associated with gain-related delta-RewP activation or loss-related theta-FRN activation. We observed a significant interaction between Outcome (Previous gain/Previous Loss) and delta-RewP activation, *t*(129.37) = −4.40, *p* < .001. This was due to a significant negative association between delta-RewP and risky gambles following gains, standardized delta-RewP fixed-effect estimate = −.29, 95% CI = [−.44 −.14], *t*(220) = −3.86, *p* < .001 (Figure 3a). There was no association between delta-RewP and risky choices following loss feedback, *p* = .99. Similarly, there was no association between theta-FRN activation and risky choices, *p* > .27. This analysis clarifies that decreased delta-band processing of gains is predictive of higher risk-taking behaviors on the gambling task, but theta-band processing of losses is not similarly predictive of risk-taking. Thus, delta and theta-band activity seem to exhibit separate associations with psychopathology and behavior that are, respectively, consistent with externalizing decision-making behaviors and with negative emotional reactivity.

## 4 Discussion

We examined gain/loss processing, posttraumatic stress, and alcohol use in previously deployed US military veterans. We found no group differences in gain/loss processing based on PTSD and alcohol dependence/abuse DSM-IV-TR categorical diagnoses. However, the intrusive reexperiencing symptom dimension was associated with enhanced frontal theta signaling during loss, indicating increased salience for negative outcomes. Concurrently, increased alcohol use was associated with reduced signaling, suggesting heavy drinking reduces neural salience related to exaggerated negative outcome perception. Heavy alcohol use and risky choices were also separately associated with decreased delta signaling during wins, consistent with the interpretation that delta-band activity reflects increased externalizing behaviors that dissociate from effects of negative emotional reactivity. Results support using dimensional measures to parse the complex clinical presentation of PTSD into elements that better map onto neural functions of salience processing and serve as intervention targets.

The anterior cingulate cortex (ACC) is a key node in the brain salience network (8) and has a central role in cognitive control (37), negative affect (9,38), and valuation (39). Mediofrontal event-related potentials in theta frequencies are believed to originate in or near the ACC (9). In particular, the theta-band FRN reflects outcome salience (40). We used the theta-FRN as a measure of neural processing of salient stimuli to investigate the impact of PTSD symptoms and alcohol use on responses to negatively valenced stimuli.

Fear extinction models propose PTSD is driven by persistent fear in situations where fear is no longer adaptive (41,42). Generalized fear may then lead to larger than expected salience responses to everyday stimuli such as simple decision-making feedback. A combination of decreased attentional control (21,43) and increased salience-related brain activation to loss feedback raises the possibility that features of PTSD are linked to a failure to control attention towards negative stimuli during a benign gambling task. In this way insufficient attentional control and emotion dysregulation could underpin maladaptive reactivity to negative outcomes as seen in the current sample of veterans. Such reactivity likely generalizes to their daily lives (44) hyperresponsiveness in the salience network. Predictive coding theories indicate that the brain makes constant predictions about sensory stimuli and minimizes prediction error by updating these predictions from experience (4). Negative future predictions are particularly intense in PTSD and contribute to enhanced processing of negative information (5,6). Thus, the association between loss-related theta and reexperiencing is predicted by multiple complementary perspectives on PTSD. Yet, our analysis of alcohol use enables differentiation between these perspectives.

Predictions vary regarding the relationship between alcohol use and brain salience signaling in individuals with posttraumatic stress. Fear extinction theories predict alcohol use could enhance salience responses because alcohol impairs fear extinction (45,46). Attentional control theories predict alcohol use could enhance salience responses by disrupting attentional control (47). Alternatively, emotional hyperreactivity accounts are consistent with negative reinforcement effects of drinking (48), predicting that increased drinking would acutely decrease the salience of negative outcomes. The effects of alcohol in lessening emotional reactivity are typically short-lived, but they may serve as the initial seeds for establishing long-term heavy alcohol use. Paradoxically, this prolonged alcohol consumption may contribute to increased negative emotional reactivity in the long-term (49,50). These predictions of heavy drinking being related to increased loss salience are inconsistent with the pattern we report, where increased drinking is linked to reduced brain loss salience signaling which is consistent with predictive coding models.

In line with predictive coding models, we put forth the interpretation that chronic alcohol use might reduce the intensity of threat predictions. This implies that alcohol use should be associated with decreased salience signaling during loss, as increasing alcohol consumption would reduce the intensity of negative priors in individuals with PTSD. Individuals with AD have lower anticipatory brain activity prior to rewards, suggesting reduced ability to make predictions (51). Our findings of decreased brain salience signaling (i.e., theta-band FRN) associated with alcohol use align with this predictive coding framework while alternative explanations such as attentional control or fear extinction theories do not fully account for the observed data. As previously noted, fear extinction and attentional control accounts predict that alcohol use should increase brain salience signaling, because alcohol use impairs both fear extinction and attentional control.

We found additional evidence of an association between blunted delta-RewP processing and heavy drinking. The RewP has a similar broad scalp topography and timing as the P300, which has been repeatedly linked with genetic risk for alcoholism (14–16). Reduced P300 amplitudes reflect externalizing personality traits (52,53), including impulsivity, aggressiveness, disinhibition, and risky or antisocial behaviors that are linked to substance use disorders and other psychopathology including antisocial personality disorder and conduct disorders (12,54). Delta-RewP amplitude was also associated with higher rates of post-gain risky choices on the gambling task, suggesting decreased reward signaling can predict riskier task behavior. Our findings parallel those of Bernat and colleagues (11), who used the same gambling paradigm and showed that delta power was related to externalizing traits and risky choice behavior. We interpret this effect as replicating prior evidence that general externalizing aspects of heavy drinking, including risky choices during the gambling task, are related to reduced brain processing of rewards. A similar brain process also appears to mark familial risk for externalizing psychopathology.

Despite informative findings there are limitations to the current investigation. The cross-sectional sample does not allow assessment of whether theta-FRN associations represent consequences or risk/vulnerability factors for posttraumatic stress (55–57). Longitudinal examinations before and after stressor exposure could help establish whether theta-FRN activation represents consequences or risk/vulnerability factors for intrusive reexperiencing. A sample of data from new military recruits prior to and following military stressors (58) using similar paradigms would be informative for this question. Longitudinal data could also be leveraged to develop adaptive reduction of theta loss signaling into a biomarker for treatment response in PTSD. We might predict that if an individual’s reexperiencing symptoms improve, their theta-FRN response to losses should also normalize. Theta-FRN normalization should precede remission of intrusive reexperiencing symptoms, as neural responses indicating losses are becoming less salient would eventually modify even very intense threat predictions. Another limitation is our focus on outcome valuation, while value-based decisions involve additional processes (59). Future studies should capture neural activation during other value-based decision-making stages such as prediction and action selection. These could include gambling paradigms with semi-predictable outcomes, such as multi-armed bandits (60). In these experimental tasks PTSD should be associated with negative predictions, while alcohol use should be associated with less intense predictions.

In sum, our study demonstrates that frontal theta evoked by losses reflects the opposing influences of intrusive reexperiencing and heavy drinking on the salience of loss. The results are supportive of recent predictive coding accounts of PTSD (5,6). When individuals chronically engage in hazardous alcohol use, the functional effect of reduced loss-related theta activity may be relief from negative future predictions, and thus from negative emotional reactivity. These results also suggest that interventions that act on frontal theta (61) might rescue some aspects of enhanced salience signaling linked to intrusive reexperiencing, a promising avenue for future computationally informed attempts to treat PTSD.

## Figures and Tables

**Table 1.** Demographic and clinical characteristics of sample.

	No PTSD						PTSD+Subthreshold					

	No AD			AD			No AD			AD		

Variable	n	M	SD	n	M	SD	n	M	SD	n	M	SD

Total Count	59			12			38			19		
Female	8			2			0			0		
Race												
White	52			12			35			19		
Black	2			0			0			0		
Asian	1			0			0			0		
Multiracial	4			0			3			0		
Age (years)		33.42	8.22		30.83	7.57		31.16	8.26		31.42	7.30
Education (years)		5.44	0.70		4.83	0.71		5.21	0.66		5.21	0.79
Depressive Disorder Diagnosis	5			3			16			9		
mTBI Experienced	19			6			21			12		
CAPS Intrusive Reexperiencing		10.22	4.62		9.14	4.53		16.76	5.79		19.37	6.29
CAPS Avoidance		3.83	2.85		4.14	3.85		8.68	3.11		9.37	3.73
CAPS Dysphoria		12.27	7.31		9.43	3.60		26.13	8.91		29.37	8.64
CAPS Hyperarousal		4.89	3.45		7.43	3.64		7.92	3.40		9.11	2.69
AUDIT-C		4.14	2.10		8.25	1.54		4.00	2.61		6.89	2.18
Above AUDIT-C Cutoff	36			12			17			17		
MN-BEST Blast mTBI Severity		1.00	1.71		0.92	1.16		2.03	3.00		1.89	2.16

PTSD = posttraumatic stress disorder, mTBI = mild traumatic brain injury, N = count, M = mean, SD = standard deviation, CAPS = Clinician-Administered PTSD Scale, AUDIT =Alcohol Use Disorders Identification Test, MN-BEST = Minnesota Blast Exposure Screening Tool. “+Subthreshold” reflects individuals who meet criteria for at least one symptom from each symptom domain of DSM-IV PTSD. The AUDIT-C cutoff was ≥ 4 for men and ≥ 3 for women.

## References

[1] Fulton JJ, Calhoun PS, Wagner HR, Schry AR, Hair LP, Feeling N, The prevalence of posttraumatic stress disorder in Operation Enduring Freedom/Operation Iraqi Freedom (OEF/OIF) Veterans: a meta-analysis. J Anxiety Disord. 2015 Apr;31:98–107. 25768399 10.1016/j.janxdis.2015.02.003

[2] Seal KH, Cohen G, Waldrop A, Cohen BE, Maguen S, Ren L. Substance use disorders in Iraq and Afghanistan veterans in VA healthcare, 2001–2010: Implications for screening, diagnosis and treatment. Drug Alcohol Depend. 2011 Jul;116(1–3):93–101. 21277712 10.1016/j.drugalcdep.2010.11.027

[3] Yufik T, Simms LJ. A meta-analytic investigation of the structure of posttraumatic stress disorder symptoms. J Abnorm Psychol. 2010;119:764–76. 21090877 10.1037/a0020981 PMC4229035

[4] Friston K, Kiebel S. Predictive coding under the free-energy principle. Philos Trans R Soc B Biol Sci. 2009 May 12;364(1521):1211–21. 10.1098/rstb.2008.0300 PMC2666703 19528002

[5] Kube T, Berg M, Kleim B, Herzog P. Rethinking post-traumatic stress disorder - A predictive processing perspective. Neurosci Biobehav Rev. 2020 Jun;113:448–60. 32315695 10.1016/j.neubiorev.2020.04.014

[6] Putica A, Felmingham KL, Garrido MI, O’Donnell ML, Van Dam NT. A predictive coding account of value-based learning in PTSD: Implications for precision treatments. Neurosci Biobehav Rev. 2022 Jul 1;138:104704. 35609683 10.1016/j.neubiorev.2022.104704

[7] Gehring WJ, Willoughby AR. The Medial Frontal Cortex and the Rapid Processing of Monetary Gains and Losses. Science. 2002 Mar 22;295(5563):2279–82. 11910116 10.1126/science.1066893

[8] Seeley WW, Menon V, Schatzberg AF, Keller J, Glover GH, Kenna H, Dissociable Intrinsic Connectivity Networks for Salience Processing and Executive Control. J Neurosci. 2007 Feb 28;27(9):2349–56. 17329432 10.1523/JNEUROSCI.5587-06.2007 PMC2680293

[9] Cavanagh JF, Shackman AJ. Frontal Midline Theta Reflects Anxiety and Cognitive Control: Meta-Analytic Evidence. J Physiol Paris. 2015;109(0):3–15. 24787485 10.1016/j.jphysparis.2014.04.003 PMC4213310

[10] Proudfit GH. The reward positivity: From basic research on reward to a biomarker for depression. Psychophysiology. 2015;52(4):449–59. 25327938 10.1111/psyp.12370

[11] Bernat EM, Nelson LD, Steele VR, Gehring WJ, Patrick CJ. Externalizing psychopathology and gain-loss feedback in a simulated gambling task: dissociable components of brain response revealed by time-frequency analysis. J Abnorm Psychol. 2011 May;120(2):352–64. 21319875 10.1037/a0022124 PMC3092030

[12] Krueger RF, Markon KE, Patrick CJ, Iacono WG. Externalizing Psychopathology in Adulthood: A Dimensional-Spectrum Conceptualization and Its Implications for DSM–V. J Abnorm Psychol. 2005 Nov;114(4):537–50. 16351376 10.1037/0021-843X.114.4.537 PMC2242352

[13] Volpe U, Mucci A, Bucci P, Merlotti E, Galderisi S, Maj M. The cortical generators of P3a and P3b: A LORETA study. Brain Res Bull. 2007 Jul 12;73(4):220–30. 17562387 10.1016/j.brainresbull.2007.03.003

[14] Benegal V, Jain S, Subbukrishna DK, Channabasavanna SM. P300 amplitudes vary inversely with continuum of risk in first degree male relatives of alcoholics. Psychiatr Genet. 1995 Winter;5(4):149. 8750356 10.1097/00041444-199524000-00001

[15] Iacono WG, Malone SM, McGue M. Substance use disorders, externalizing psychopathology, and P300 event-related potential amplitude. Int J Psychophysiol. 2003 May 1;48(2):147–78. 12763572 10.1016/s0167-8760(03)00052-7

[16] Polich J, Bloom FE. P300, Alcoholism Heritability, and Stimulus Modality. Alcohol. 1999 Feb;17(2):149–56. 10064383 10.1016/s0741-8329(98)00047-0

[17] Kamarajan C, Rangaswamy M, Tang Y, Chorlian DB, Pandey AK, Roopesh BN, Dysfunctional reward processing in male alcoholics: An ERP study during a gambling task. J Psychiatr Res. 2010 Jul 1;44(9):576–90. 20035952 10.1016/j.jpsychires.2009.11.019 PMC2878886

[18] Lieberman L, Gorka SM, Funkhouser CJ, Shankman SA, Phan KL. Impact of posttraumatic stress symptom dimensions on psychophysiological reactivity to threat and reward. J Psychiatr Res. 2017 Sep 1;92:55–63. 28410485 10.1016/j.jpsychires.2017.04.002 PMC10593111

[19] Davenport ND, Lim KO, Sponheim SR. White matter abnormalities associated with military PTSD in the context of blast TBI. Hum Brain Mapp. 2014 Nov 12;36(3):1053–64. 25387950 10.1002/hbm.22685 PMC6869802

[20] First MB, Gibbon M. The Structured Clinical Interview for DSM-IV Axis I Disorders (SCID-I) and the Structured Clinical Interview for DSM-IV Axis II Disorders (SCID-II). In: Comprehensive handbook of psychological assessment, Vol 2: Personality assessment. Hoboken, NJ, US: John Wiley & Sons, Inc.; 2004. p. 134–43.

[21] Marquardt CA, Pokorny VJ, Disner SG, Nelson NW, McGuire KA, Sponheim SR. Inefficient Attentional Control Explains Verbal-Memory Deficits Among Military Veterans With Posttraumatic Reexperiencing Symptoms. Clin Psychol Sci. 2022 May 1;10(3):499–513. 10.1177/21677026211025018 PMC10663645 38020495

[22] Blake DD, Weathers FW, Nagy LM, Kaloupek DG, Gusman FD, Charney DS, The development of a Clinician-Administered PTSD Scale. J Trauma Stress. 1995 Jan;8(1):75–90. 7712061 10.1007/BF02105408

[23] Weathers FW, Keane TM, Davidson JRT. Clinician-administered PTSD scale: A review of the first ten years of research. Depress Anxiety. 2001;13(3):132–56. 11387733 10.1002/da.1029

[24] Palmieri PA, Weathers FW, Difede J, King DW. Confirmatory factor analysis of the PTSD Checklist and the Clinician-Administered PTSD Scale in disaster workers exposed to the World Trade Center Ground Zero. J Abnorm Psychol. 2007;116:329–41. 17516765 10.1037/0021-843X.116.2.329

[25] Simms LJ, Watson D, Doebbelling BN. Confirmatory factor analyses of posttraumatic stress symptoms in deployed and nondeployed veterans of the Gulf War. J Abnorm Psychol. 2002;111:637–47. 12428777 10.1037//0021-843x.111.4.637

[26] Marquardt CA, Pokorny VJ, Kang SS, Cuthbert BN, Sponheim SR. Posttraumatic stress symptom dimensions and brain responses to startling auditory stimuli in combat veterans. J Abnorm Psychol. 2021;130:455–67. 34472883 10.1037/abn0000552 PMC11772048

[27] Saunders JB, Aasland OG, Babor TF, de la Fuente JR, Grant M. Development of the Alcohol Use Disorders Identification Test (AUDIT): WHO Collaborative Project on Early Detection of Persons with Harmful Alcohol Consumption--II. Addict Abingdon Engl. 1993 Jun;88(6):791–804. 10.1111/j.1360-0443.1993.tb02093.x 8329970

[28] Nelson NW, Hoelzle JB, McGuire KA, Ferrier-Auerbach AG, Charlesworth MJ, Sponheim SR. Neuropsychological evaluation of blast-related concussion: Illustrating the challenges and complexities through OEF/OIF case studies. Brain Inj. 2011 May 1;25(5):511–25. 21405948 10.3109/02699052.2011.558040

[29] Gratton G, Coles MG, Donchin E. A new method for off-line removal of ocular artifact. Electroencephalogr Clin Neurophysiol. 1983 Apr;55(4):468–84. 6187540 10.1016/0013-4694(83)90135-9

[30] Bernat EM, Williams WJ, Gehring WJ. Decomposing ERP time–frequency energy using PCA. Clin Neurophysiol. 2005 Jun 1;116(6):1314–34. 15978494 10.1016/j.clinph.2005.01.019

[31] Buzzell GA, Niu Y, Aviyente S, Bernat E. A practical introduction to EEG Time-Frequency Principal Components Analysis (TF-PCA). Dev Cogn Neurosci. 2022 Jun 1;55:101114. 35636345 10.1016/j.dcn.2022.101114 PMC9156873

[32] Jeong J, Williams WJ. Kernel design for reduced interference distributions. IEEE Trans Signal Process. 1992 Feb;40(2):402–12.

[33] Koller M. robustlmm: An R Package for Robust Estimation of Linear Mixed-Effects Models. J Stat Softw. 2016 Dec 6;75:1–24. 32655332 10.18637/jss.v075.i01 PMC7351245

[34] Arnau J, Bendayan R, Blanca MJ, Bono R. The effect of skewness and kurtosis on the robustness of linear mixed models. Behav Res Methods. 2013 Sep 1;45(3):873–9. 23299397 10.3758/s13428-012-0306-x

[35] Bates D, Mächler M, Bolker B, Walker S. Fitting Linear Mixed-Effects Models Using lme4. J Stat Softw. 2015 Oct 7;67(1):1–48.

[36] Lenth RV, Buerkner P, Giné-Vázquez I, Herve M, Jung M, Love J, emmeans: Estimated Marginal Means, aka Least-Squares Means [Internet]. 2022 [cited 2022 Nov 7]. Available from: https://CRAN.R-project.org/package=emmeans

[37] Carter CS. Anterior Cingulate Cortex, Error Detection, and the Online Monitoring of Performance. Science. 1998 May 1;280(5364):747–9. 9563953 10.1126/science.280.5364.747

[38] Shackman AJ, Salomons TV, Slagter HA, Fox AS, Winter JJ, Davidson RJ. The integration of negative affect, pain and cognitive control in the cingulate cortex. Nat Rev Neurosci. 2011 Mar;12(3):154–67. 21331082 10.1038/nrn2994 PMC3044650

[39] Shenhav A, Botvinick MM, Cohen JD. The expected value of control: an integrative theory of anterior cingulate cortex function. Neuron. 2013 Jul 24;79(2):217–40. 23889930 10.1016/j.neuron.2013.07.007 PMC3767969

[40] Talmi D, Atkinson R, El-Deredy W. The Feedback-Related Negativity Signals Salience Prediction Errors, Not Reward Prediction Errors. J Neurosci. 2013 May 8;33(19):8264–9. 23658166 10.1523/JNEUROSCI.5695-12.2013 PMC6619637

[41] Duits P, Cath DC, Lissek S, Hox JJ, Hamm AO, Engelhard IM, Updated Meta-Analysis of Classical Fear Conditioning in the Anxiety Disorders. Depress Anxiety. 2015;32(4):239–53. 25703487 10.1002/da.22353

[42] Zuj DV, Palmer MA, Lommen MJJ, Felmingham KL. The centrality of fear extinction in linking risk factors to PTSD: A narrative review. Neurosci Biobehav Rev. 2016 Oct 1;69:15–35. 27461912 10.1016/j.neubiorev.2016.07.014

[43] Schoorl M, Putman P, Van Der Werff S, Van Der Does AJW. Attentional bias and attentional control in Posttraumatic Stress Disorder. J Anxiety Disord. 2014 Mar 1;28(2):203–10. 24291395 10.1016/j.janxdis.2013.10.001

[44] Morey RA, Dolcos F, Petty CM, Cooper DA, Hayes JP, LaBar KS, The role of trauma-related distractors on neural systems for working memory and emotion processing in posttraumatic stress disorder. J Psychiatr Res. 2009 May;43(8):809–17. 19091328 10.1016/j.jpsychires.2008.10.014 PMC2684984

[45] Holmes A, Fitzgerald PJ, MacPherson KP, DeBrouse L, Colacicco G, Flynn SM, Chronic alcohol remodels prefrontal neurons and disrupts NMDAR-mediated fear extinction encoding. Nat Neurosci. 2012 Oct;15(10):1359–61. 22941108 10.1038/nn.3204 PMC3471649

[46] Smiley CE, McGonigal JT, Nimchuk KE, Gass JT. Optogenetic manipulation of the prelimbic cortex during fear memory reconsolidation alters fear extinction in a preclinical model of comorbid PTSD/AUD. Psychopharmacology (Berl). 2021 Nov 1;238(11):3193–206. 34347171 10.1007/s00213-021-05935-3

[47] Goldstein RZ, Volkow ND. Dysfunction of the prefrontal cortex in addiction: neuroimaging findings and clinical implications. Nat Rev Neurosci. 2011 Nov;12(11):652–69. 22011681 10.1038/nrn3119 PMC3462342

[48] Koob GF. Negative reinforcement in drug addiction: the darkness within. Curr Opin Neurobiol. 2013 Aug 1;23(4):559–63. 23628232 10.1016/j.conb.2013.03.011

[49] Jacobsen LK, Southwick SM, Kosten TR. Substance use disorders in patients with posttraumatic stress disorder: a review of the literature. Am J Psychiatry. 2001 Aug;158(8):1184–90. 11481147 10.1176/appi.ajp.158.8.1184

[50] Stewart SH, Pihl RO, Conrod PJ, Dongier M. Functional associations among trauma, ptsd, and substance-related disorders. Addict Behav. 1998 Nov 1;23(6):797–812. 9801717 10.1016/s0306-4603(98)00070-7

[51] Luijten M, Schellekens AF, Kühn S, Machielse MWJ, Sescousse G. Disruption of Reward Processing in Addiction : An Image-Based Meta-analysis of Functional Magnetic Resonance Imaging Studies. JAMA Psychiatry. 2017 Apr 1;74(4):387–98. 28146248 10.1001/jamapsychiatry.2016.3084

[52] Gilmore CS, Malone SM, Bernat EM, Iacono WG. Relationship between the P3 event-related potential, its associated time-frequency components, and externalizing psychopathology. Psychophysiology. 2010;47(1):123–32. 19674392 10.1111/j.1469-8986.2009.00876.x PMC2860032

[53] Patrick CJ, Bernat EM, Malone SM, Iacono WG, Krueger RF, McGue M. P300 amplitude as an indicator of externalizing in adolescent males. Psychophysiology. 2006 Jan;43(1):84–92. 16629688 10.1111/j.1469-8986.2006.00376.x PMC2242347

[54] Patrick CJ, Drislane LE. Triarchic Model of Psychopathy: Origins, Operationalizations, and Observed Linkages with Personality and General Psychopathology. J Pers. 2015 Dec;83(6):627–43. 25109906 10.1111/jopy.12119

[55] Bonanno GA. Resilience in the Face of Potential Trauma. Curr Dir Psychol Sci. 2005 Jun 1;14(3):135–8.

[56] Luthar SS, Cicchetti D, Becker B. The Construct of Resilience: A Critical Evaluation and Guidelines for Future Work. Child Dev. 2000;71(3):543–62. 10953923 10.1111/1467-8624.00164 PMC1885202

[57] Polusny MA, Erbes CR, Kramer MD, Thuras P, DeGarmo D, Koffel E, Resilience and Posttraumatic Stress Disorder Symptoms in National Guard Soldiers Deployed to Iraq: A Prospective Study of Latent Class Trajectories and Their Predictors. J Trauma Stress. 2017;30(4):351–61. 28763565 10.1002/jts.22199

[58] Polusny MA, Marquardt CA, Campbell EH, Filetti CR, Noël VV, Disner SG, Advancing Research on Mechanisms of Resilience (ARMOR) Longitudinal Cohort Study of New Military Recruits: Results from a Feasibility Pilot Study. Res Hum Dev. 2021;18(3):212–29. 34887706 10.1080/15427609.2021.1964898 PMC8651241

[59] Rangel A, Camerer C, Montague PR. A framework for studying the neurobiology of value-based decision making. Nat Rev Neurosci. 2008 Jul;9(7):545–56. 18545266 10.1038/nrn2357 PMC4332708

[60] O’Doherty JP, Dayan P, Friston K, Critchley H, Dolan RJ. Temporal Difference Models and Reward-Related Learning in the Human Brain. Neuron. 2003 Apr 24;38(2):329–37. 12718865 10.1016/s0896-6273(03)00169-7

[61] Chiang HS, Motes M, Kraut M, Vanneste S, Hart J. High-definition transcranial direct current stimulation modulates theta response during a Go-NoGo task in traumatic brain injury. Clin Neurophysiol. 2022 Nov 1;143:36–47. 36108520 10.1016/j.clinph.2022.08.015 PMC10545365

